# Supplementary material for: Bayesian refinement of protein structures and ensembles against SAXS data using molecular dynamics
Source: PLoS Comput Biol. 2017 Oct 18;13(10):e1005800. doi: 10.1371/journal.pcbi.1005800 (PMC5662244; doi:10.1371/journal.pcbi.1005800)
Supplement: S1 Archive — (BZ2) [file pcbi.1005800.s011.tar.bz2 › gromacs_4.62_bayesian_saxs_refi/share/html/online/g_analyze.html]

g\_analyze


| g\_analyze Main Table of Contents |

**VERSION 4.6  
Sat 19 Jan 2013**

---

### Description

g\_analyze reads an ASCII file and analyzes data sets.
A line in the input file may start with a time
(see option -time) and any number of y-values may follow.
Multiple sets can also be
read when they are separated by & (option -n);
in this case only one y-value is read from each line.
All lines starting with # and @ are skipped.
All analyses can also be done for the derivative of a set
(option -d).

All options, except for -av and -power, assume that the
points are equidistant in time.

g\_analyze always shows the average and standard deviation of each
set, as well as the relative deviation of the third
and fourth cumulant from those of a Gaussian distribution with the same
standard deviation.

Option -ac produces the autocorrelation function(s).
Be sure that the time interval between data points is
much shorter than the time scale of the autocorrelation.

Option -cc plots the resemblance of set i with a cosine of
i/2 periods. The formula is:  
2 (integral from 0 to T of y(t) cos(i π t) dt)^2 / integral from 0 to T of y^2(t) dt  
This is useful for principal components obtained from covariance
analysis, since the principal components of random diffusion are
pure cosines.

Option -msd produces the mean square displacement(s).

Option -dist produces distribution plot(s).

Option -av produces the average over the sets.
Error bars can be added with the option -errbar.
The errorbars can represent the standard deviation, the error
(assuming the points are independent) or the interval containing
90% of the points, by discarding 5% of the points at the top and
the bottom.

Option -ee produces error estimates using block averaging.
A set is divided in a number of blocks and averages are calculated for
each block. The error for the total average is calculated from
the variance between averages of the m blocks B\_i as follows:
error^2 = sum (B\_i - **)^2 / (m\*(m-1)).
These errors are plotted as a function of the block size.
Also an analytical block average curve is plotted, assuming
that the autocorrelation is a sum of two exponentials.
The analytical curve for the block average is:  
f(t) = σ\*sqrt(2/T ( α (τ\_1 ((exp(-t/τ\_1) - 1) τ\_1/t + 1)) +  
(1-α) (τ\_2 ((exp(-t/τ\_2) - 1) τ\_2/t + 1)))),  
where T is the total time.
α, τ\_1 and τ\_2 are obtained by fitting f^2(t) to error^2.
When the actual block average is very close to the analytical curve,
the error is σ\*sqrt(2/T (a τ\_1 + (1-a) τ\_2)).
The complete derivation is given in
B. Hess, J. Chem. Phys. 116:209-217, 2002.

Option -bal finds and subtracts the ultrafast "ballistic"
component from a hydrogen bond autocorrelation function by the fitting
of a sum of exponentials, as described in e.g.
O. Markovitch, J. Chem. Phys. 129:084505, 2008. The fastest term
is the one with the most negative coefficient in the exponential,
or with -d, the one with most negative time derivative at time 0.
-nbalexp sets the number of exponentials to fit.

Option -gem fits bimolecular rate constants ka and kb
(and optionally kD) to the hydrogen bond autocorrelation function
according to the reversible geminate recombination model. Removal of
the ballistic component first is strongly advised. The model is presented in
O. Markovitch, J. Chem. Phys. 129:084505, 2008.

Option -filter prints the RMS high-frequency fluctuation
of each set and over all sets with respect to a filtered average.
The filter is proportional to cos(π t/len) where t goes from -len/2
to len/2. len is supplied with the option -filter.
This filter reduces oscillations with period len/2 and len by a factor
of 0.79 and 0.33 respectively.

Option -g fits the data to the function given with option
-fitfn.

Option -power fits the data to b t^a, which is accomplished
by fitting to a t + b on log-log scale. All points after the first
zero or with a negative value are ignored.

Option -luzar performs a Luzar & Chandler kinetics analysis
on output from g\_hbond. The input file can be taken directly
from g\_hbond -ac, and then the same result should be produced.

### Files

| option | filename | type | description |
| --- | --- | --- | --- |
| **-f** | graph.xvg | Input | xvgr/xmgr file |
| **-ac** | autocorr.xvg | Output, Opt. | xvgr/xmgr file |
| **-msd** | msd.xvg | Output, Opt. | xvgr/xmgr file |
| **-cc** | coscont.xvg | Output, Opt. | xvgr/xmgr file |
| **-dist** | distr.xvg | Output, Opt. | xvgr/xmgr file |
| **-av** | average.xvg | Output, Opt. | xvgr/xmgr file |
| **-ee** | errest.xvg | Output, Opt. | xvgr/xmgr file |
| **-bal** | ballisitc.xvg | Output, Opt. | xvgr/xmgr file |
| **-g** | fitlog.log | Output, Opt. | Log file |

### Other options

| option | type | default | description |
| --- | --- | --- | --- |
| **-[no]h** | bool | no | Print help info and quit || **-[no]version** | bool | no | Print version info and quit || **-nice** | int | 0 | Set the nicelevel || **-[no]w** | bool | no | View output .xvg, .xpm, .eps and .pdb files || **-xvg** | enum | xmgrace | xvg plot formatting: xmgrace, xmgr or none || **-[no]time** | bool | yes | Expect a time in the input || **-b** | real | -1 | First time to read from set || **-e** | real | -1 | Last time to read from set || **-n** | int | 1 | Read this number of sets separated by & || **-[no]d** | bool | no | Use the derivative || **-bw** | real | 0.1 | Binwidth for the distribution || **-errbar** | enum | none | Error bars for -av: none, stddev, error or 90 || **-[no]integrate** | bool | no | Integrate data function(s) numerically using trapezium rule || **-aver\_start** | real | 0 | Start averaging the integral from here || **-[no]xydy** | bool | no | Interpret second data set as error in the y values for integrating || **-[no]regression** | bool | no | Perform a linear regression analysis on the data. If -xydy is set a second set will be interpreted as the error bar in the Y value. Otherwise, if multiple data sets are present a multilinear regression will be performed yielding the constant A that minimize χ^2 = (y - A\_0 x\_0 - A\_1 x\_1 - ... - A\_N x\_N)^2 where now Y is the first data set in the input file and x\_i the others. Do read the information at the option -time. || **-[no]luzar** | bool | no | Do a Luzar and Chandler analysis on a correlation function and related as produced by g\_hbond. When in addition the -xydy flag is given the second and fourth column will be interpreted as errors in c(t) and n(t). || **-temp** | real | 298.15 | Temperature for the Luzar hydrogen bonding kinetics analysis (K) || **-fitstart** | real | 1 | Time (ps) from which to start fitting the correlation functions in order to obtain the forward and backward rate constants for HB breaking and formation || **-fitend** | real | 60 | Time (ps) where to stop fitting the correlation functions in order to obtain the forward and backward rate constants for HB breaking and formation. Only with -gem || **-smooth** | real | -1 | If this value is >= 0, the tail of the ACF will be smoothed by fitting it to an exponential function: y = A exp(-x/τ) || **-filter** | real | 0 | Print the high-frequency fluctuation after filtering with a cosine filter of this length || **-[no]power** | bool | no | Fit data to: b t^a || **-[no]subav** | bool | yes | Subtract the average before autocorrelating || **-[no]oneacf** | bool | no | Calculate one ACF over all sets || **-acflen** | int | -1 | Length of the ACF, default is half the number of frames || **-[no]normalize** | bool | yes | Normalize ACF || **-P** | enum | 0 | Order of Legendre polynomial for ACF (0 indicates none): 0, 1, 2 or 3 || **-fitfn** | enum | none | Fit function: none, exp, aexp, exp\_exp, vac, exp5, exp7, exp9 or erffit || **-ncskip** | int | 0 | Skip this many points in the output file of correlation functions || **-beginfit** | real | 0 | Time where to begin the exponential fit of the correlation function || **-endfit** | real | -1 | Time where to end the exponential fit of the correlation function, -1 is until the end |

---

http://www.gromacs.org  
gromacs@gromacs.org**
